# Life-long sports engagement enhances adult erythrocyte adenylate energetics

**DOI:** 10.1038/s41598-021-03275-y

**Published:** 2021-12-09

**Authors:** Barbara Pospieszna, Krzysztof Kusy, Ewa Maria Slominska, Jacek Zieliński

**Affiliations:** 1 Department of Athletics, Strength and Conditioning, Poznan University of Physical Education, Królowej Jadwigi 27/39, 61-871 Poznan, Poland; 2 Department of Biochemistry, Medical University of Gdansk, Dębinki 1, 80-211 Gdansk, Poland

**Keywords:** Physiology, Biomarkers, Health care

## Abstract

Regular physical activity reduces age-related metabolic and functional decline. The energy stored in adenine nucleotides (ATP, ADP, and AMP) is essential to enable multiple vital functions of erythrocytes and body tissues. Our study aimed to predict the rate of age-related changes in erythrocyte adenylate energetics in athletes and untrained controls. The erythrocyte concentration of adenylates was measured in 68 elite endurance runners (EN, 20–81 years), 58 elite sprinters (SP, 21–90 years), and 62 untrained individuals (CO, 20–68 years). Resting concentrations of ATP, total adenine nucleotide pool, and ADP/AMP ratio were lowest in the CO group and highest in the SP group. The concentration of erythrocyte ADP and AMP was lowest in the EN group and highest in the CO group. In all studied groups, we found a significant increase in the concentration of most erythrocyte adenylate metabolites with age. For ADP and AMP, the trend was also significant but decreasing. Our study strongly suggests that lifelong sports and physical activity participation supports erythrocyte energetics preservation. Although the direction and the predicted rates of change are similar regardless of the training status, the concentrations of particular metabolites are more advantageous in highly trained athletes than in less active controls. Of the two analyzed types of physical training, sprint-oriented training seems to be more efficient in enhancing erythrocyte metabolism throughout adulthood and old age than endurance training.

## Introduction

Red blood cell (RBC) energy metabolism is unique compared to other human cells because erythrocytes are incapable to synthesize purine nucleotides in the de novo pathway and must synthesize these compounds in salvage reactions by reutilizing purine bases and nucleosides^1^. The synthesis of adenosine triphosphate (ATP) in RBC relies solely on the anaerobic conversion of glucose by the Embden-Meyerhof pathway due to the lack of nuclei and mitochondria in matured RBCs^2^. The energy stored in ATP and other adenine nucleotides (adenosine diphosphate, ADP; adenosine monophosphate, AMP) is essential to preserve multiple vital functions of erythrocytes, such as maintaining glycolysis and purine metabolism, electrolyte gradient between plasma and cytoplasm, glutathione synthesis, asymmetry of phospholipid membrane, and keeping iron in hemoglobin (Hb) in the ferrous state^2^. Several factors limit the erythrocyte energy status, particularly by reducing ATP concentration, adenosine triphosphate/adenosine diphosphate ratio (ATP/ADP), and adenylate energy charge (AEC). These are RBC enzymopathies^2^, decreased erythrocyte deformability^3,4^, sedentary lifestyle^5,6^, neurodegenerative disorders, e.g. Alzheimer’s disease^7^, or metabolic disorders, e.g. diabetes^8^. There is, however, very scarce information about the impact of aging on the erythrocyte energy status.

The rate of age-dependent biological deterioration vastly varies between individuals due to lifestyle, environmental, genetic, or epigenetic factors. The age-dependent decline in physiological functional capacity includes the decline in muscle strength and function^9^ as well as aerobic and anaerobic capacity in both trained and untrained individuals, with lower levels in the latter group^10,11^.

However, there is evidence that, apart from natural aging, the rate of aerobic capacity decline is strongly related to the individual rate of physical activity reduction^12,13^.

Blood cells—showing alterations in the expression of several markers of immunosenescence, inflammation, and oxidative stress with advancing age—are frequently used to present the inevitable aging-related changes at the tissue level^3,4,14,15^, yet only limited scientific data is available on the age-related changes in erythrocyte energetics^16,17^. In our previous study, we observed that plasma purine concentration (hypoxanthine, uric acid, xanthine) increased with advancing age, indicating a depletion of the skeletal muscle adenine nucleotide pool. We also revealed that in highly trained athletes an increase in hypoxanthine–guanine phosphoribosyltransferase (HGPRT) activity compensated for this disadvantageous effect, suggesting that lifelong training supports the salvage pathway of the adenylate pool restoration^18^.

The associations between increased blood volume, improved blood composition, and various forms of physical activity were reported on several occasions^19^. Compared to untrained counterparts, athletes usually had a higher proportion of reticulocytes and mature erythrocytes, thus higher hemoglobin content and mean corpuscular Hb concentration^20–22^. Exercise training elevates the concentration of hormones (e.g. erythropoietin, catecholamines, cortisol, testosterone, growth hormone, and insulin-like growth factor) that, in turn, stimulate RBC production and their release from the bone marrow^19,21,23^. It also modulates the hematopoietic bone marrow activity^19,24^. High-intensity exercise causing profound homeostasis disturbances leads to increased post-exercise hemolysis and subsequent restitution of erythropoiesis^21,25–27^. Participation in exercise training triggers considerable modifications of erythrocyte membrane properties towards higher membrane stability and lower osmotic fragility^15,22^. Therefore, a long-lasting training regime can induce the increment in RBC turnover, resulting in a shorter RBC lifespan (e.g. ~ 40% shorter in endurance runners than in sedentary controls) and a higher proportion of younger RBCs in trained individuals^15,21,28^. Having a higher proportion of young RBCs—characterized by greater oxygen affinity, deformation ability, osmotic resistance, a higher rate of glycolysis, and better antioxidant protection^29^—athletes have enhanced exercise capacity and higher erythrocyte energy status compared to recreationally trained and sedentary individuals^5,6,21^. We found in young adults that periodized endurance and sprint training, but not recreational activity, resulted in a significant increment in erythrocyte adenylate energy potential during a one-year training cycle^6^.

Given the above considerations, this study aimed to evaluate the levels and the predicted rates of age-related changes in erythrocyte adenylate energetics in endurance- and sprint-trained athletes compared to untrained controls. We hypothesized that (i) the level of the erythrocyte concentration of ATP, total adenylate pool (TAN), and adenylate energy charge are higher in athletes compared to untrained controls regardless of age, and (ii) the level and the rate of the age-related decline in erythrocyte energetics depend on the type of lifelong training model.

## Material and methods

### Participants

Two hundred and six healthy non-smoking physically fit men, aged 20–90 years, participated in this study. They were divided into three groups according to their level of sports engagement and sports discipline. The first group comprised of 86 endurance runners (EN; long- and middle-distance runners; 20–81 years) and the second group of 58 speed-power or sprint-trained athletes (SP; sprinters, specialized in sprint running, jumping, and combined events; age range 21–90 years). The control group consisted of 62 untrained individuals (CO; 20–68 years). In the age gap of 20 to 39 years, there were 27 participants in the EN group, 19 in SP, and 25 in the CO group. In age gaps 40–59 and 60–79 years of age there were 42 and 15 endurance runners, 23 and 11 sprinters, and 24 and 13 controls. In the oldest group (> 80 years) there were two endurance athletes and five sprinters. The inclusion criteria were (i) no reported history of a cardiovascular/cardiopulmonary disease or other severe/chronic diseases, (ii) no major orthopedic injury or illness resulting in an inability to run, (iii) no medications that could affect circulatory function, (iv) normal resting electrocardiogram, (v) body mass index (BMI) below 30.0 kg m^−2^, (vi) normal RBC count and Hb content, and (vii) no pathological states known of significantly elevated adenylate pool and concentration of ATP, e.g. sickle cell disease, diabetes, leukemia, sepsis, tuberculosis, meningococcal infection, or renal insufficiency. Apart from a health interview, all participants underwent a preliminary clinical examination focused on cardiovascular, pulmonary, and musculoskeletal systems, with blood count analysis to detect possible hematological diseases.

The additional inclusion criteria into athletic groups were either the membership of the Polish national track and field team (athletes under 35 years of age were included in the study within a routine periodical physiological evaluation) or the regular participation in masters European and world championships. All master athletes were the leaders in their age categories (ranked 1–10 in European and world championships) and were volunteers recruited by the agency of national teams managers, via leaflets, and personal communication. To ensure a maximal level of sports performance and to reduce the effect of seasonal variation in training load and physical capacity, all examinations were performed during the competition phase of the annual training cycle.

Before the study, data on training history (years of participation in competitive sport) and current training volume (average hours per week over the past year) were collected. On average, EN athletes were involved in competitive sport for 26.7 ± 14.4 years (from 6 to 66 years depending on age; the relationship between training history and age: r = 0.92, p < 0.001) and trained weekly for 9.1 ± 3.5 h week^−1^ (range: 5‒18.5 h week^−1^ depending on age; r = − 0.73, p < 0.001). SP athletes participated in sports competition for 28.6 ± 18.8 years, ranging from 7 to 77 years depending on age (r = 0.93, p < 0.001) and trained weekly for 8.7 ± 3.2 h week^−1^ (range: 5‒15 h week^−1^ depending on age; r = − 0.70, p < 0.001). There were no significant differences in the above training characteristics between the athletic groups.

The controls were invited to participate in the study through announcements via local mass media. They recreationally practiced popular forms of physical activity (e.g., jogging, swimming, team games) during their leisure time, but did not exceed 150 min of moderate-intensity physical activity per week recommended for maintaining health by the World Health Organization^30^.

The aim of the research, testing methodology and potential risks were explained, and written informed consent was obtained from each participant. The study was approved by the Local Bioethical Committee at the Karol Marcinkowski Poznan University of Medical Sciences. The study was retrospectively registered in the clinical trials registry (Clinicaltrials.gov) under the trial registration number: NCT05113914. All testing procedures were carried out in the Human Movement Laboratory of the Poznan University of Physical Education (Poznan, Poland) following the relevant guidelines and regulations.

### Somatic and physiological variables

Participants were asked to avoid training sessions, competition, and other heavy exertion for at least 24 h before the study. They arrived at the laboratory in the morning after a 12-h overnight fast. Body mass (kg) and height (cm) were measured using a digital stadiometer (Seca 285, SECA, Hamburg, Germany) with the participants only wearing underwear and without footwear. Body mass index (BMI) was later calculated according to the formula: BMI = weight (kg)/height^2^ (m^2^).

Maximal oxygen uptake (${\dot{V}}$ ˙O_2_max) was measured two hours after a light standardized breakfast (bread and butter, water, without coffee or tea). An incremental running test was conducted on the treadmill (h/p/cosmos Sports & Medical GmbH, Nussdorf—Traunstein, Germany). Initially, the exercise protocol included standing still for 3 min and walking for 3 min at a constant speed of 4 km/h. After this introductory part, the speed was increased to 8 km/h and then every 3 min by 2 km/h until the volitional exhaustion was reached. Oxygen consumption was measured with the use of a portable breath-by-breath ergospirometer (MetaMax 3B-R2, Cortex Biophysics GmbH, Leipzig, Germany). All data was transmitted wirelessly to a Windows-based computer, then recorded and analyzed using Meta Soft Studio 5.1.0 software (Cortex Biophysik GmbH, Leipzig, Germany). Heart rate (HR) was recorded continuously using the Polar Bluetooth Smart H6 monitor (Polar Electro, Oy, Kempele, Finland). ${\dot{V}}$O_2_max was expressed in ml kg^−1^ min^−1^ (body mass-adjusted values). ${\dot{V}}$O_2_max was considered achieved if at least three of the following criteria were met: (i) a plateau in ${\dot{V}}$O_2_ despite an increase in running speed, (ii) blood lactate (LA) concentration ≥ 9 mmol/l, (iii) respiratory exchange ratio ≥ 1.10, and (iv) HR ≥ 95% of the age-predicted maximum^31^.

### Blood sampling and measurements

Venous blood samples were taken twice from an antecubital vein: at rest and 5 min after exercise. Blood samples were collected into two separate tubes: ethylenediaminetetraacetic acid (EDTA) (2.7 ml) and lithium heparinate (4.9 ml) as an anticoagulant (S-monovette, Sarstedt, Nümbrecht, Germany). The first tube was used for the determination of hemoglobin concentration, mean corpuscular hemoglobin concentration (MCHC), and hematocrit (HCT) value. All these measurements were carried out in 10 μl of aspirated blood using the 18-parametric automated hematology analyzer Mythic® 18 (Orphée, Geneva, Switzerland). Immediately before erythrocytes were separated from plasma, 20 μL of the whole blood from the second tube was used to assay LA concentration by the spectrophotometric enzymatic method (Biosen C-line, EKF Diagnostics, Barleben, Germany). Within 3 min after drawing a sample, the whole blood in the second tube was centrifuged (5000 rpm, 5 min, 37 °C; the Universal 320 R centrifuge, Hettich Lab Technology, Tuttlingen, Germany). The plasma and buffy coat were removed and the erythrocytes were washed three times with the buffered 0.9% sodium chloride (NaCl) solution and centrifuged each time (3000 rpm, 5 min, 4 °C). After a final wash, the resulting erythrocyte pellet was resuspended with a small volume of phosphate-buffered saline (PBS) and immediately deep-frozen at − 80 °C until the analysis of erythrocyte purine nucleotides (ATP, ADP, AMP). The procedure of the erythrocyte purine nucleotides sampling and analysis was described in detail in our previous paper^6^. The measurements were performed using high-performance liquid chromatography (HPLC) with UV–Vis detection (Merck-Hitachi/Agilent, Japan/USA) according to the method described in detail by Slominska et al.^32^. The values of ATP/ADP, ADP/AMP, total adenine nucleotide pool (TAN = ATP + ADP + AMP) and AEC = ([ATP] + 0.5 [ADP])/([ATP] + [ADP] + [AMP]) were later calculated.

### Statistics

All statistical analyses were performed using Statistica 13.3 software (StatSoft, Inc., Tulsa, OK). The sample size was a priori estimated based on the assumption that the effect size will be at least medium. Given the α-level of 0.05 and the statistical power (1 − β) of 0.80, it was a priori calculated that 36 participants in a single group would be needed to detect significant differences and correlations (G*Power software; Heinrich-Heine-Universität, Düsseldorf, Germany). The Shapiro–Wilk test confirmed the normal distribution of analyzed variables, therefore further analyses were performed using parametric tests. To estimate longitudinal relationships between age and erythrocyte purine metabolites, linear regression analyses were performed. A one-way analysis of variance (ANOVA) with the post-hoc Bonferroni test was used to determine differences between studied groups. The level of significance was set at p < 0.05. The statistical power of ANOVA analyses varied from 0.16 (age) to 1.0 for descriptive and exercise characteristics (resting heart rate, ${\dot{V}}$˙O_2_max, and covered distance) and was 1.00 for all erythrocyte purine nucleotides.

## Results

All values are presented as mean ± standard deviation (SD). The somatic, exercise, and hematological characteristics of study participants are shown in Table 1. Mean age did not significantly differ between groups. There were significant between-group differences in body mass (SP and CO were heavier than EN), body height (CO were higher than EN), and body mass index (SP had higher BMI than EN). Athletic groups did not differ in terms of resting heart rate, measured both at rest and immediately after maximal exercise, but they differed from controls who achieved significantly higher values. Maximal oxygen uptake differed between the groups and was smallest in the control group and highest in endurance-trained athletes. Compared to controls, endurance athletes had higher values of Hb_rest_, Hct_rest_, and LA_rest_. We observed significantly higher concentrations of MCHC_rest_ in sprinters than in controls. Only the resting lactate concentration was significantly different between the athletic groups.

**Table 1:** Descriptive, exercise and hematological characteristics of endurance runners (EN; n = 86), sprinters (SP; n = 58), and controls (CO; n = 62).

Variable	EN	SP	CO	p	Effect size (η^2^)
Age	46.4 ± 15.2	47.3 ± 19.1	44.1 ± 15.1	0.527	0.006
BM (kg)	72.4 ± 7.0^†,‡^	77.6 ± 8.4^#^	76.0 ± 6.1^#^	< 0.001*	0.090
BH (cm)	177.3 ± 5.6^‡^	179.4 ± 8.2	180.0 ± 5.1^#^	0.024*	0.036
BMI (kg/m^2^)	23.0 ± 2.0^†^	24.1 ± 1.7^#^	23.5 ± 1.4	0.003*	0.057
HR_rest_ (bpm)	69.2 ± 3.9^‡^	70.5 ± 3.4^‡^	74.4 ± 4.3^#,†^	< 0.001*	0.237
HR_max_ (bpm)	177.0 ± 11.0^‡^	178.3 ± 10.4^‡^	183.8 ± 10.6^#,†^	0.001*	0.070
${\dot{V}}$O_2max_ (ml/kg/min)	58.6 ± 8.6^†,‡^	48.2 ± 8.5^#,‡^	41.7 ± 5.6^#,†^	< 0.001*	0.468
Hb_rest_ (g/l)	15.4 ± 1.0^‡^	15.2 ± 0.8	14.9 ± 0.7^#^	0.005*	0.052
HCT_rest_	0.45 ± 0.03	0.44 ± 0.02	0.44 ± 0.02	0.041*	0.031
MCHC_rest_ (g/dl)	34.4 ± 2.1	34.8 ± 1.6^‡^	33.9 ± 1.4^†^	0.014*	0.041
LA_rest_ (mmol/l)	1.2 ± 0.4^†,‡^	1.0 ± 0.3^#^	1.2 ± 0.3^#^	0.001*	0.070

Data are given as means ± SD; *Significant between-group differences as indicated by one-way ANOVA (p < 0.05), ^†^significantly different from sprint-trained athletes, ^‡^significantly different from untrained controls, ^#^significantly different from endurance-trained athletes.

*BM* body mass, *BH* body height, *BMI* body mass index, *HR* heart rate, ${\dot{V}}$*˙O*_*2*_*max* maximal oxygen uptake, *Hb* hemoglobin, *HCT* hematocrit, *MCHC* mean corpuscular hemoglobin, *LA* plasma lactate concentration; *rest* resting value, *max* maximal value.

As shown in Table 2, all analyzed erythrocyte purine metabolites differed significantly between the three groups. Resting concentrations of ATP, TAN level, and ADP/AMP ratio were lowest in the CO group, higher in the EN group, and highest in the SP group. The concentration of erythrocyte ADP and AMP was lowest in the EN group, higher in the SP group, and highest in the CO group. Endurance-trained athletes had the highest values of AEC and ATP/ADP ratio with lower values in the SP and lowest in the CO group. There were no significant differences between EN and CO. Only the concentration of ADP was different between SP and CO groups. Significant differences were found in most adenylate pool indices between the athletic groups, except for AMP and AEC values.

**Table 2:** Concentration of erythrocyte adenylate pool of endurance runners (EN; n = 86), sprinters (SP; n = 58), and controls (CO; n = 62).

Variable (µmol/L RBC)	EN	SP	CO	p	Effect size (η^2^)
ATP_rest_	1736.3 ± 98.8^†,‡^	1779.0 ± 97.1^#,‡^	1647.1 ± 85.8^#,†^	< 0.001*	0.234
ADP_rest_	215.2 ± 23.4^†,‡^	240.8 ± 26.6^#^	243.5 ± 23.7^#^	< 0.001*	0.232
AMP_rest_	22.0 ± 4.3^‡^	20.64 ± 4.0^‡^	26.8 ± 4.3^#,†^	< 0.001*	0.269
TAN_rest_	1973.4 ± 96.2^†,‡^	2040.4 ± 83.5^#,‡^	1917.4 ± 77.5^#,†^	< 0.001*	0.227
AEC_rest_	0.934 ± 0.01^‡^	0.931 ± 0.01^‡^	0.922 ± 0.01^#,†^	< 0.001*	0.239
ATP/ADP_rest_	8.2 ± 1.0^†,‡^	7.5 ± 1.0^#,‡^	6.8 ± 0.9^#,†^	< 0.001*	0.251
ADP/AMP_rest_	10.0 ± 1.5^†,‡^	11.9 ± 1.4^#,‡^	9.2 ± 1.4^#,†^	< 0.001*	0.350

Data are given as means ± SD. *Significant between-group differences as indicated by one-way ANOVA (p < 0.05); ^†^Significantly different from sprint-trained athletes. ^‡^Significantly different from untrained controls. ^#^Significantly different from endurance-trained athletes.

*ATP* adenosine-5′-triphosphate, *ADP* adenosine-5′-diphosphate, *AMP* adenosine-5′-monophosphate, *TAN* total adenine nucleotides, *AEC* adenylate energy charge, *ATP/ADP* adenosine-5′-triphosphate/adenosine-5′-diphosphate ratio, *ADP/AMP* adenosine-5′-diphosphate/ adenosine-5′-monophosphate ratio. TAN (total adenine nucleotides) was calculated from following formula: TAN = [ATP] + [ADP] + [AMP], AEC (adenylate energy charge) was evaluated according to the formula by Atkinson AEC = ([ATP] + 0.5[ADP])/([ATP] + [ADP] + [AMP]), *rest* resting value.

In all three groups, the majority of variables showed a significant decrease with age as predicted by linear regression. Medium to large effect size of relationships with age (p ≤ 0.001) was achieved for HR_max_, ${\dot{V}}$O_2_max, distance covered during the exercise test, and Hb_rest_ (r^2^ = 0.23 − 0.82).

**Figure 1:**
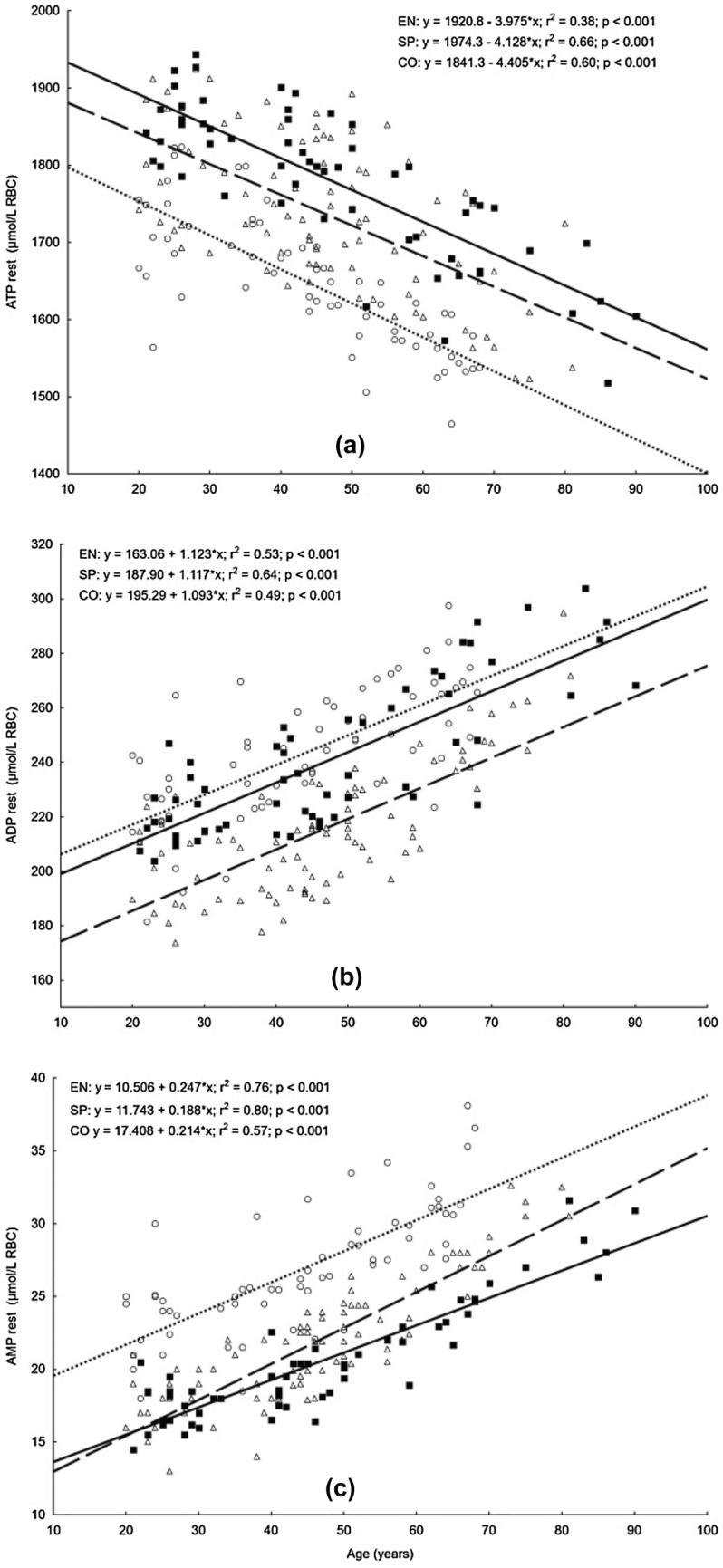
Relationships between age and erythrocyte concentration of ATP, ADP, and AMP at rest (**a**, **b**, **c**) in endurance runners (open triangle, dashed lines; n = 86), sprinters (filled square, solid lines; n = 58) and controls (open circle, dotted lines; n = 62). *ATP* adenosine-5′-triphosphate, *ADP* adenosine-5′-diphosphate, *AMP* adenosine-5′-monophosphate, *rest* resting value.

**Figure 2:**
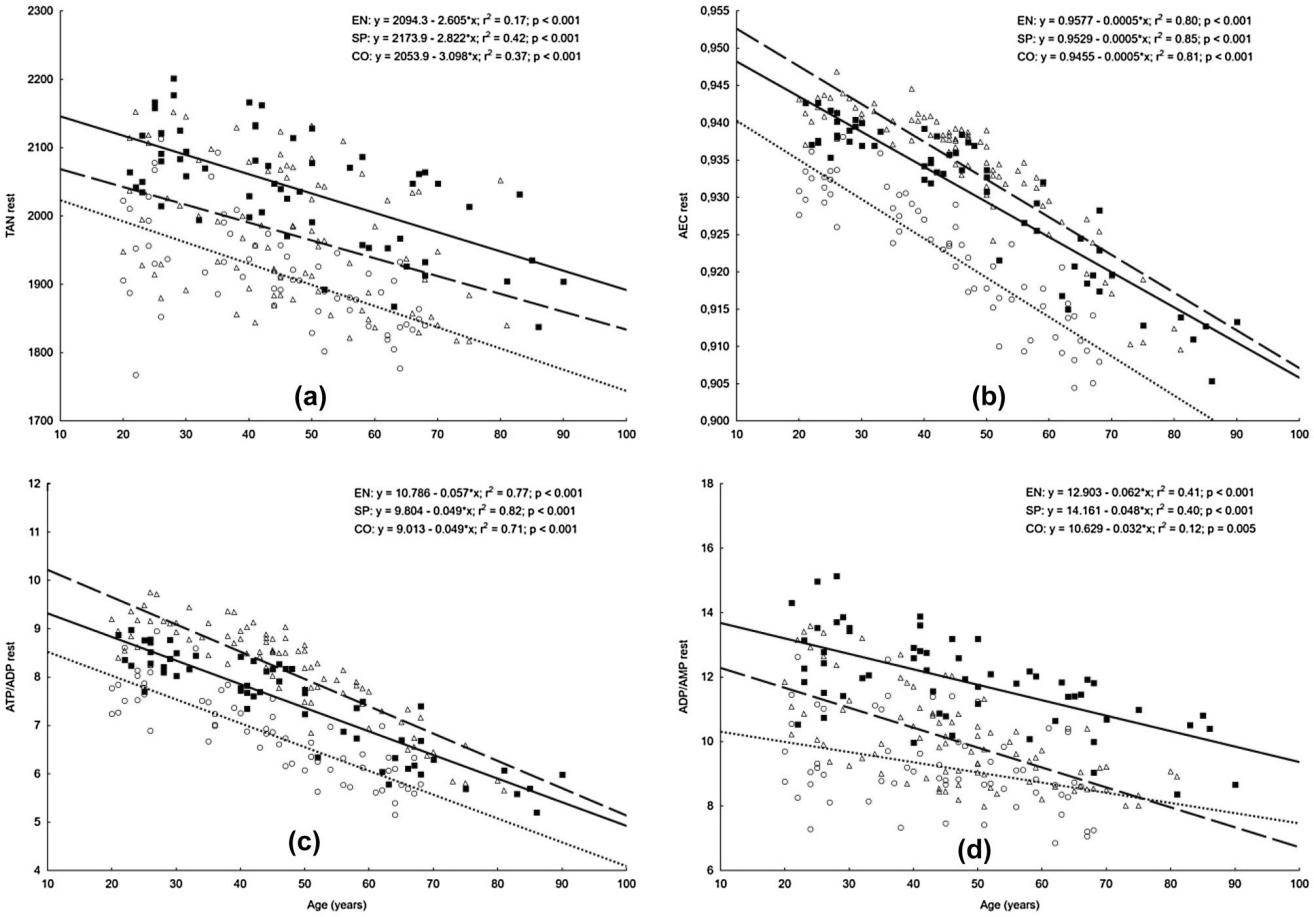
Relationships between age and the level of erythrocyte TAN, AEC, ATP/ADP, and ADP/AMP at rest (**a**, **b**, **c**, **d**) in endurance runners (open triangle, dashed lines; EN; n = 86), sprinters (filled square, solid lines; SP; n = 58) and controls (open circle, dotted lines; CO; n = 62). *TAN* total adenine nucleotides (calculated from formula: TAN = [ATP] + [ADP] + [AMP]), *AEC* adenylate energy charge (evaluated according to Atkinson’s formula: AEC = ([ATP] + 0.5[ADP])/([ATP] + [ADP] + [AMP])), *ATP/ADP* adenosine-5′-triphosphate/adenosine-5′-diphosphate ratio, *ADP/AMP* adenosine-5′-diphosphate/ adenosine-5′-monophosphate ratio, *rest* resting value.

The linear relationships between age and concentrations of erythrocyte adenylate metabolites are shown in Figs. 1 and 2. In all studied groups, a significant increase in all variables was observed, except for the resting concentrations of ADP and AMP. The highest coefficients of determination (r^2^ > 0.55) were noted for AMP, AEC, and ATP/ADP in both athletic groups, and only in the sprint group for resting ATP and ADP values. In athletic groups, the smallest coefficients of determination (r^2^ < 0.25) were shown for TAN. Among controls, the highest coefficients of determination were noted in measurements for ATP, AMP and AEC, and ATP/ADP, and the smallest for ADP/AMP.

## Discussion

There are three main findings of this study. (i) The direction and magnitude of the age-related trends in the resting concentration of erythrocyte adenylate metabolites  are similar in both athletes and controls. The significant decrements in the adenylate pool (ATP, TAN, AEC, ATP/ADP, and ADP/AMP) and increases in the concentration of ADP and AMP are expected with aging, suggesting a gradual deterioration in RBC energetics and their metabolic efficiency. (ii) Athletic groups showed a higher concentration of ATP and values of TAN, AEC, ATP/ADP, and ADP/AMP, but lower concentrations of ADP and AMP compared to untrained participants, regardless of age. (iii) Also, the lifelong picture of erythrocyte energetics seems to be somewhat more advantageous in sprint-trained athletes (higher ATP and TAN, with similar AEC values) compared to endurance-trained runners, which is consistent with our previous observation in young athletes^6^.

Research on erythrocyte energetics and concentration of RBC adenylate metabolites with advancing age is scarce. Kosenko et al.^16^ analyzed the differences in the level of antioxidant status and RBC energy state between patients with Alzheimer's disease, non-Alzheimer dementia, age-matched controls, and younger adult controls. They showed a significant age-related decline in erythrocyte ATP, TAN, and AEC levels, with no differences between older groups, suggesting that dementia does not affect the level of erythrocyte energetics. In contrast, Chaleckis et al.^17^ considered erythrocyte ATP concentration as not related to aging. The main interest of their study was the evaluation of 126 blood metabolites of which 55 were RBC-enriched. In the blood samples of 30 volunteers—15 young (29 ± 4 years of age) and 15 elderly (81 ± 7 years of age)—they found only fourteen blood compounds that showed age-related dependence. They only found that the erythrocyte concentration of ATP and most of the adenine nucleotides had coefficients of variation at a low level (< 0.4), suggesting that they support fundamental RBC functions.

In this study, we analyzed the predicted change in indices of erythrocyte adenylate energy metabolism with advancing age in a large group of 206 healthy males. We have found medium to strong correlations between age and the concentration of erythrocyte adenylates, regardless of training status. Our results imply that in healthy males the energetic condition of erythrocytes gradually decreases between 20 and 90 years of age.

In young and healthy individuals, well-developed erythrocytes function at their maximal efficiency, so the balance between RBC production and apoptosis is preserved, and erythrocyte total adenylate pool and adenylate energy charge are high and stable. With advancing age, the natural erythrocyte antioxidant protection of enzymatic and non-enzymatic origin, which should counteract the appearing reactive oxygen species, becomes less effective^7,16,26,27,33–35^. Therefore, as humans age, their blood cells become less efficient, mainly because of the changes in cell membrane structure and function^3,4,36,37^. These changes contribute to the enhanced RBC dehydration, aggregation, and thrombosis^38^. At the same time, higher rates of macrocytes and megalocytes^39^, but a decreased quantity of reticulocytes leads to the age-related increased prevalence of anisocytosis in the elderly^33^. Although we did not study the detailed underlying mechanisms, all mentioned causes may contribute to some extent to the progressive age-related deterioration in RBC energetics shown in this study. Yet, it should be emphasized that the predicted changes in erythrocyte energetics, though statistically significant, did not exceed the reference values for healthy humans. For example, the decreased AEC—which is known for its regulatory influence on every level of coupling between processes that provide, consume or store energy in erythrocytes—did not exceed the reference values ranging from 0.85 to 0.95^36^ even in the oldest control participants (lowest AEC = 0.904).

In our previous study, we showed improved erythrocyte energy status in young athletes compared to age-matched recreationally trained controls^6^. Similar findings were also reported by Dudzińska et al^5^. The novel findings of the present study are higher ATP concentration, TAN pool, AEC, ATP/ADP and ADP/AMP ratios, concurrent with lower concentrations of ADP and AMP in athletes compared to healthy controls across the whole age range. The significant differences between athletes and healthy controls observed at the age of 20 (baseline values) indicate an early training-induced adaptation in erythrocyte adenylate energetic efficiency. In our study, the tendencies of gradual deterioration in RBC energetics and their metabolic efficiency remained very similar between groups across decades. It seems that in healthy males, constantly active in sports, the baseline level of erythrocyte adenylate energetics (achieved at the age of 20) may determine the levels for the rest of life. Our observation is in line with the results of ten- and twenty-year follow-ups of maximum oxygen uptake level in master track athletes^12,13^. The longitudinal observation proved that there are significant differences in the rate of ${\dot{V}}$O_2_max decline depending on the individual level of sports involvement during aging. The highest decline was noted for athletes who greatly reduced training intensity and the smallest for ones who remained elite.

The most probable explanation of the between-group differences in adenylate pool and RBC energy charge is the difference in the rate of RBC turnover in trained vs. untrained subjects. Physical training is known to modulate the hematopoietic bone marrow activity by stimulating its hyperplasia, improving the hematopoietic microenvironment, or increasing the rate of proliferation and maturation of hematopoietic stem cells^19,24^. Moreover, high-intensity exercise leads to profound homeostasis disturbances (oxidative stress, local hypoxia, acidification, hypoglycemia, or hyperthermia) and, consequently, to increased post-exercise hemolysis and subsequent restitution of erythropoiesis^21,25–27^.

The erythrocyte lifespan in healthy adults is approximately 120 days ± 15% and remains quite constant throughout human life^40–42^. Undertaking additional physical activity is proved to shorten RBC lifespan and increase RBC turnover^21,25,41,43^. Walking 30‒50 km at moderate intensity on four consecutive days is enough to generate a functionally improved red blood cell population with higher deformability and a decreased tendency to aggregate in 66-years-old volunteers^43^. It seems that sudden exercise-induced stress causes a shift towards the younger RBC population by accelerating the senescence of the oldest, most vulnerable, red blood cells^43^. A prolonged engagement in physical training stabilizes the shortened RBC lifespan. Weight et al.^41^ demonstrated that the RBC lifespan is 67.23 ± 22.2 and 72.4 3 ± 26.0 days in endurance-trained men and women compared to 113.4 ± 31.0 and 114.1 ± 29.0 days, respectively, in non-exercising individuals. The enhanced training-induced erythropoiesis, together with concurrently increased hemolysis of the oldest cells, was shown to cause beneficial hemorheological changes (e.g. greater proportion of young RBCs, increased RBC deformability, higher membrane stability, higher antioxidant defence) that can contribute to an increased tissue oxygen supply^15,19,21–24,28^ and higher metabolic efficiency of erythrocytes in trained individuals.

Our study also aimed to evaluate whether the changes in erythrocyte adenylate energetics are dependent on the type of the adopted training model. Considering only the level of AEC, which was not significantly different between sprinters and endurance-trained runners, we may conclude that the type of training is irrelevant to the exercise-induced enhancement of erythrocyte energetic efficiency. Nevertheless, the significantly higher levels of TAN and higher concentrations of ATP and ADP in sprinters, persisting across the whole analyzed age range, suggest a higher energetic potential of the adenylate pool in speed-power athletes. Frequently repeated maximal-intensity exercise loads stimulate rapid energy demand, possibly leading to increased adenylate pool and ATP concentration in erythrocytes to stabilize the RBC energy charge. Given that the erythrocyte TAN pool vastly depends on the balance between the rate of AMP synthesis (from adenine and adenosine) and its degradation^36,44^, it is likely that sprint training shifts this balance towards diminished erythrocyte AMP degradation and preserved AMP production. It is also possible that during maximal exercise the disbalance between the inner and outer side of the RBC membrane and the highly activated transport ATPases impose additional stress on energy metabolism^36,37^, resulting in further adaptation of RBC.

A large sample of 206 healthy males aged 20‒90 years and the inclusion of two groups of highly-trained athletes specializing in two different sport disciplines are the strengths of this study. The main limitation is that we did not measure the activity of enzymes involved in specific metabolic pathways in RBC^2^. The combined assessment of enzymatic activity and erythrocyte concentration of adenylates would allow a deeper insight into the mechanisms of RBC energy metabolism. It would also be of interest to broaden the study with younger male and female individuals differing in the levels of habitual physical activity^45^ and with patients with diseases known of significantly elevated adenylate pool and concentration of ATP, e.g. sickle cell disease or diabetes^36^. Inclusion of younger individuals would allow (i) to indicate the approximate age, at which the training-induced adaptation in erythrocyte adenylate energetic peaks in ontogenesis, and (ii) to determine at what stage of the sport carrier the differences in erythrocyte energetics arise between athletes of different sports specializations. The involvement of patients would allow distinguishing between the rates of age-related changes in erythrocyte adenylate energetics in healthy and pathological aging. Some longitudinal observations revealed that there are significant differences in the rate of maximum oxygen uptake decline depending on the individual level of sports involvement during aging^12,13^. It would be of interest to include former athletes and clarify whether the rate of RBC energetics decline is different between athletes who substantially reduced their training loads and those who remained elite. Future research could also cover the analysis of the influence of some other confounding factors, e.g. lifestyle factors, genetics, diet or environment, possibly affecting the level of RBC energetics.

In conclusion, our study strongly suggests that sports participation support erythrocyte energetics preservation in humans across a very wide age range. Although the direction and the predicted rates of change are similar regardless of training status, the concentrations of particular metabolites remain more advantageous in highly-trained athletes than in less active controls. Of the two analyzed types of physical training, sprint-oriented training seems to be more efficient in enhancing erythrocyte metabolism throughout adulthood and old age than endurance training.

## Data Availability

The datasets generated and/or analyzed during the current study are available from the corresponding author on reasonable request.
